# Power Law Behaviour in Complex Systems

**DOI:** 10.3390/e20090671

**Published:** 2018-09-05

**Authors:** António M. Lopes, J. A. Tenreiro Machado

**Affiliations:** 1 UISPA–LAETA/INEGI, Faculty of Engineering, University of Porto, R. Dr. Roberto Frias, 4200–465 Porto, Portugal; 2 Institute of Engineering, Polytechnic of Porto, Department of Electrical Engineering, R. Dr. António Bernardino de Almeida, 431, 4249–015 Porto, Portugal

**Keywords:** power law, dynamics, complex systems, fractional calculus, entropy, information theory

Complex systems (CS) are ubiquitous in many areas, including financial markets, highway transportation networks, telecommunication networks, world and country economies, social networks, immunological systems, living organisms, computational systems, and electrical and mechanical structures. CS are characterized by a global dynamics much richer than the dynamics of their composing individual parts. Power law statistics is the most common description of complex dynamics. Since power law statistical distributions and fractional dynamics are connected, fractional order dynamics is also expected to occur in CS.

The Special Issue focuses on original and new research results on systems dynamics in science and engineering. It includes 10 manuscripts addressing novel issues and specific topics that illustrate the role of entropy and information theory in CS. In the follow-up, the selected manuscripts are presented in alphabetical order.

In the paper “A Simple and Adaptive Dispersion Regression Model for Count Data”, Hadeel Klakattawi, Veronica Vinciotti, and Keming Yu propose a discrete Weibull regression model for different types of dispersion that occur in count datasets. The authors show that the maximum likelihood can be used for efficient parameter estimation. The description of the model, parameter inference, and model diagnostics is accompanied by simulated and real data analyses [1].

The paper “Complexity Analysis of Global Temperature Time Series” by António M. Lopes and J. A. Tenreiro Machado studies global temperature time series (TTS) by means of four different complexity indices, namely the Lempel–Ziv complexity, sample entropy, signal harmonics power ratio, and fractal dimension. In a first phase, the monthly mean TTS is pre-processed using empirical mode decomposition, and the TTS trend is calculated. In a second phase, the complexity of the detrended signals is estimated. The four indices capture distinct features of the TTS dynamics in a four-dimensional space. Hierarchical clustering is adopted for dimensional reduction and visualization in the two-dimensional space. The results show that TTS complexity exhibits space-time variability, suggesting the presence of distinct climate forcing processes in both dimensions. Numerical examples with real-world data demonstrate the effectiveness of the approach [2].

In the paper “Criterion of Existence of Power-Law Memory for Economic Processes” by Vasily Tarasov and Valentina Tarasova, different criteria for the existence of power-law type memory in economic processes are proposed. These criteria allow the use of fractional calculus to construct dynamic models of economic processes. They can also be used to identify linear integro-differential operators that can be considered as fractional derivatives and integrals of non-integer orders. The suggested criteria are illustrated in various examples [3].

In “Diffusion on Middle-ξ Cantor Sets”, Alireza Khalili Golmankhaneh, Arran Fernandez, Ali Khalili Golmankhaneh, and Dumitru Baleanu study $C^{\xi }$-calculus on generalized Cantor sets, with self-similar properties and fractional dimensions that exceed their topological dimensions. The authors suggest a calculus on the middle-ξ Cantor sets for 0<ξ<1. Differential equations on the middle-ξ Cantor sets are solved, and results are presented for illustrative examples. The conditions for super-, normal, and sub-diffusion on fractal sets are given [4].

In “Finite Difference Method for Time-Space Fractional Advection-Diffusion Equations with Riesz Derivative”, Sadia Arshad, Dumitru Baleanu, Jianfei Huang, Maysaa Mohamed Al Qurashi, Yifa Tang, and Yue Zhao present a numerical scheme to solve the time-space fractional advection-diffusion equation, where the Riesz and the Caputo derivatives are considered in spatial and temporal directions, respectively. The Riesz space derivative is approximated by the second-order fractional weighted and shifted Grünwald–Letnikov formula. The proposed scheme is formally proved with the second-order accuracy in both space and time. Numerical experiments are presented to illustrate the theoretical analysis [5].

The manuscript “Hierarchical Scaling in Systems of Natural Cities” by Yanguang Chen and Bin Jiang analyses the power law behaviors in systems of natural cities by reconstructing the urban hierarchy with cascade structure. Cities of the U.S.A., Britain, France, and Germany are taken as examples to perform empirical calculations. The hierarchical scaling relations can be well fitted to the data points within the scaling ranges of the number, size, and area of the natural cities. The size-number and area-number scaling exponents are close to 1, and the size-area allometric scaling exponent is slightly less than 1. The principle of entropy maximization of urban evolution is then employed to explain the hierarchical scaling laws, and different entropy maximizing processes are used to interpret the scaling exponents [6].

In the work “Is Natural Language a Perigraphic Process? The Theorem about Facts and Words Revisited”, Łukasz Dębowski investigate natural language. The authors demonstrate the theorem about facts and words, stating that the number of probabilistic or algorithmic facts that can be inferred from a text drawn from a process must be roughly smaller than the number of distinct word-like strings detected in it by means of the prediction by partial matching compression algorithm. They also observe that the number of word-like strings for a sample of plays by Shakespeare follows an empirical stepwise power law, in stark contrast to Markov processes. Hence, they conjecture that natural language considered as a process is not only non-Markov, but also perigraphic [7].

The paper “Non-Linear Diffusion and Power Law Properties of Heterogeneous Systems: Application to Financial Time Series” by Miguel A. Fuentes discusses the possibility of obtaining not only an anomalous diffusion process, but also a nonlinear diffusion equation that leads to a probability distribution when using a set of non-Markovian processes. That probability distribution shows a power law behavior, and reflects the anomalous transport characteristics of the ensemble of particles. The theoretical findings are illustrated when applied to a financial time series [8].

In “The Power Law Characteristics of Stock Price Jump Intervals: An Empirical and Computational Experimental Study”, by Hongduo Cao, Hui Ouyang, Ying Li, Xiaobin Li, and Ye Chen, the authors study the power law characteristics of stock price jump intervals. The classical jump-diffusion model is described as the jump-diffusion model with power law (JDMPL). An artificial stock market is designed in which an agent’s investment strategies are considered to create a dynamically changing environment. Analysis results indicate that the JDMPL effectively characterizes the stock price jumps in the market. The results also support the hypothesis that the time interval of stock price jumps is consistent with the power law, and indicate that the diversity and dynamic changes of agents’ investment strategies are the reasons for the discontinuity in the changes of stock prices [9].

In the paper “Time-Fractional Diffusion with Mass Absorption in a Half-Line Domain due to Boundary Value of Concentration Varying Harmonically in Time”, by Yuriy Povstenko and Tamara Kyrylych, the authors study the time-fractional diffusion equation with mass absorption in a half-line domain under the Dirichlet boundary condition varying harmonically in time. The Caputo derivative is employed. The solution is obtained using the Laplace transform with respect to time and the sin-Fourier transform with respect to the spatial coordinate. The results of numerical calculations are illustrated graphically [10].

In conclusion, the guest editors hope that the selected papers will help scholars and researchers to push forward the progress in the emerging area of CS and fractional dynamics.
